# Insufficiently supported in handling responsibility and demands: Findings from a qualitative study of newly graduated nurses

**DOI:** 10.1111/jocn.15483

**Published:** 2020-09-21

**Authors:** Anna Willman, Kaisa Bjuresäter, Jan Nilsson

**Affiliations:** ^1^ Department of Health Sciences Karlstad University Karlstad Sweden; ^2^ Department of Health Promotion Sciences Sophiahemmet University Stockholm Sweden

**Keywords:** acute care, competencies, new graduate nurse, nursing care

## Abstract

**Aims and objectives:**

To explore newly graduated registered nurses' experiences and how they manage complex patient situations.

**Background:**

Newly graduated registered nurses' working in acute care hospital settings are challenged by managing complex patient situations in rapidly changing clinical contexts involving increased patient acuity, comorbidities and staffing shortages.

**Design:**

Qualitative study design.

**Methods:**

Data were collected using focus groups interviews of a total of 16 newly graduated registered nurses with clinical work experience of 6 months of direct patient care in an acute care hospital setting. Analyses were conducted using qualitative content analysis. COREQ reporting guidelines were used.

**Results:**

The analysis resulted in the overarching theme “Not being sufficiently prepared and supported to meet responsibilities and demands.” The theme included three categories: “Responsibility is not in proportion to competence,” “Lack of medical competence and experience complicates patient safety” and “Strives for control to manage and organise nursing care.”

**Conclusion:**

The results show that newly graduated registered nurses' are not sufficiently supported for the level of responsibility and the demands placed on them when providing nursing in complex patient situations in acute care hospital settings. If they are given sole responsibility for multiple complex patient situations, patient safety may be compromised.

**Relevance to practice:**

Special attention need to be paid to NGRNs support to medical competence in the areas of assessing, planning, prioritizing, leading, and distributing nursing care in daily clinical settings for at least their first year of professional work.

**What does this paper contribute to the wider global clinical community?**

- Insufficient clinical competence in nursing care in complex patient patients situations among newly graduated registered nurses' (NGRNs) will threaten quality of care and patient safety. If NGRNs being made solely responsible for the nursing care of multiple complex situations is considered to compromise patient safety.
- Since the clinical competence of NGRNs is lacking in regard to take on the responsibility required in complex patient situations, introductory programmes, continuous competence development and collegial support are of crucial importance.
- NGRNs need the support of experienced RNs when working in complex patient situations. Special attention need to be paid to their medical competence in the areas of assessing, planning, prioritising, leading and distributing nursing care in daily clinical settings for at least their first year of professional work.

## 1 INTRODUCTION

Making newly graduated registered nurses (NGRNs) responsible for nursing in complex patient situations in acute care hospital settings is associated with increased patient acuity and deteriorating patient health. This is common as numbers of patients admitted to hospitals with comorbidities and multisystem disorder are increasing (Sturmberg & Lanham, 2014) due to the ageing population (Zwijsen, Nieuwenhuizen, Maarsingh, Depla, & Hertogh, 2016) and decreasing patient length of stay in hospitals (Buchan, O'may, & Dussault, 2013). Further, new medical interventions and technology are evolving at a higher rate than ever (Buchan et al., 2013). There is also a shortage of registered nurses (RNs) (ICN, 2019; WHO, 2018) and in particular experienced RNs (National Board of Health & Welfare, 2015). Due to this, NGRNs are expected to provide a level of nursing care in complex patient situations similar to that expected of experienced RNs (Purling & King, 2012), despite reports of NGRNs finding providing adequate nursing care challenging (Gardiner & Sheen, 2016).

## 2 BACKGROUND

The initial period in a RN's career can be a time of growth and development, but it can also be a time of vulnerability (Duchscher, 2009). NGRNs have reported experiencing stress, anxiety, fear of failure and incompetence during their initial time as RNs (Arrowsmith, Lau‐Walker, Norman, & Maben, 2016; Walton, Lindsay, Hales, & Rook, 2018). From the perspective of NGRNs, the assessment of patients' needs is fragmented (Benner, 2001). According to Benner's nursing theory, novice is characterised by NGRNs' difficulties in handling new situations due to a lack of experience (Benner, 2001). This means that NGRNs need regulations, such as task lists and clear guidelines, that can guide their decisions and actions regarding the provision of nursing care (Benner, 2001). Advanced beginners have learned how to handle situations based on experience. The competent stage is obtained when an RN can handle a range of different situation. A proficient RN perceives situations holistically rather than in fragments and can perceive the significance of a situation as a whole. NGRNs defined themselves as novices or advanced beginners during their first two years of practice (Benner, 2001). Clinical competence in nursing is a holistic and dynamic process that requires individual characteristics such as motivation, critical thinking, experience, attitudes, pedagogical factors, knowledge, skills and functional tasks, which leads to overall competence in nursing (Calzone, Jenkins, Culp, Caskey, & Badzek, 2014; Curuso, Fida, Sili, & Arrigoni, 2016; Notarnicola et al., 2016; Pijl‐Zieber, Barton, Konkin, Awosoga, & Caine, 2014). Clinical competence in nursing practice is the ability to apply both theoretical and practical knowledge to perform a task with a desirable outcome under various clinical contextual conditions (Benner, 2001). Nursing in complex patient situations requires critical thinking skills in order to identify changes in patients' conditions so adequate nursing interventions can be made (Shoulders, Follett, & Eason, 2014).

The complexity of patient situations is specific and dynamic where different factors interact with each other, related to different factors such as personal, social and clinical (Shippee, Shah, May, Mair, & Montori, 2012) as well as instability, variability and uncertainty (Huber, Kleinknecht‐Dolf, Kugler, & Spirig, 2020). Instability refers to unexpected events such as sudden deterioration, variability is caused by different types of health problems, comorbidity and is influenced by age, and uncertainty is affected by the diversity of health problems the patient is experiencing, patient knowledge and patients' ability to manage their health conditions. These factors are further influenced by assessment and interventions that RNs are carrying out or are not carrying out. NGRNs may have difficulty recognising changes and deterioration in patients' conditions due to lacking a holistic approach (Purling & King, 2012). NGRNs have expressed that patients with multiple diagnoses were outside their scope of expertise, and patients who experience a sudden deterioration in their condition are challenging (Gellerstedt, Moquist, Roos, Bergkvist, & Craftman, 2019). Nursing interventions and decision‐making processes in complex patient situations need to include professional judgement based on experience in addition to the use of nursing care plans and guidelines (Huber et al., 2020). In this study, nursing in complex patient situations refers to the instability, variability and uncertainty of a patient's medical treatment and everyday life, and this complexity is further increased by the nursing decisions and interventions provided. A shortage of RNs will have an impact on NGRNs as they will be challenged with new demands and responsibilities earlier and with reduced support from experienced RNs. In addition, clinical context where nursing care is carried out is changing rapidly (Lima, Newall, Kinney, Jordan, & Hamilton, 2014). Further, research on NGRNs' competence in an increasingly complex healthcare system with acute care hospital settings is needed (Kleinknecht‐Dolf et al., 2015). In order to provide high‐quality care and increase patient safety in complex patient situations, it is important to understand how NGRNs experience and handle these situations. The aim of this study was to explore NGRNs' experiences and management of complex patient situations.

## 3 METHOD

This study has an inductive qualitative design to gain a deeper understanding of the participants´ experience of a phenomenon (Polit & Beck, 2021). Data were collected through focus group interviews (FGI). The FGIs were semistructured interactive discussions that can be used among participants sharing homogeneous experiences to explore their views and opinions and are suitable for gaining understanding into complex behaviours and motivations (Morgan, 1997). The participants answered questions individually, talked and interacted with each other, and the FGIs were designed to get insights, views and explore NGRNs experiences about how they manage nursing in complex patients' situations. COREQ was used as reporting guidelines (see File S1) in line with EQUATOR (Tong, Sainsbury, & Craig, 2007).

### 3.1 Participants and setting

A total of 16 NGRNs voluntarily agreed to participate in the study, all 16 were female, and the age ranged from 22–33 years (mean age 24.6). A convenience sampling was used (Polit & Beck, 2021). Fifty‐two NGRNs employed by a county council at acute care hospitals in central Sweden participated in a mandatory clinical development programme organised by the county council held over twelve days in their first year of employment. At their first day of the clinical development programme, an introduction meeting was arranged and the NGRNs who were interested in participating in the study wrote down their contact details. Out of the 52, there were 20 NGRNs who reported interest to voluntary participate in the study, 18 of them confirmed thereafter via telephone that they wanted to participate in a FGI. Two participants were prevented from participating due to the time schedule did not work with the rest of the participants in the FGIs. The participants had six months of clinical work experience from direct patient care at different wards including medical, surgical, emergency, gynaecological, psychiatric and oncology wards. Oral and written information about the study was given to NGRNs at the first meeting of this clinical development programme.

### 3.2 Data collection

Data were collected through focus group interviews (FGI), as described by Morgan (1997). In this study, four FGIs were conducted with five participants in one group, four in two groups and three in one group. The first author acted as a moderator during these interview sessions. An assistant moderator took notes and gave an oral summery at the conclusion of each interview. As a starting point for the discussion, participating NGRNs were asked to describe their understanding of nursing in complex patient situations in relation to instability, variability and uncertainty. The same semistructured interview guide was used in all four FGIs. The interview guide contained a broad opening question: “Can you please tell us what it is like to be a newly graduated nurse?”, followed by the key question: “Can you please tell us about your experience of nursing care in complex patient situations?” and “Can you please tell us about how you manage nursing care in complex patient situations?” Probing questions were used when needed, such as: “Can you please tell me more about that?”. The interviews lasted for 62–75 min and were recorded and transcribed verbatim.

### 3.3 Data analysis

Data were analysed with a text‐driven, interpretive qualitative manifest and latent content analysis (Krippendorff, 2018). The interviews were read through repeatedly to reach an understanding of general substance and then read closely to identify manifest meaning units in each interview. The next step was to take out codes from the meaning units in each interview. Thereafter, the codes from all four interviews were used to generate subcategories based on similarities and differences, with the ability to trace each individual code back to the original text. In the next step, the subcategories were sorted into latent interpreted content in categories. Subcategories were built on manifest data, and thereafter, interpretations could be made, and the methodological process of going back and forth in the text to find different levels of abstraction to increase the ability to see context and patterns was followed (Krippendorff, 2018). From the categories, the latent content emerge represented in a theme. When the analyses were complete, the data were read in relation to the categories. The relevance of the result was finally verified by the correlation between the aim and the categories (Krippendorff, 2018). The first and second author coded two of the interviews independently, which resulted in high congruity. All authors took active part throughout every step in the analysis process. All steps in the analysis process were characterised by flexibility, verification of the original text and each step of the analysis were discussed within the research group, and any discrepancies were discussed until consensus was reached (Table 1).

**TABLE 1:** Shows examples of the analysis process and subcategories, categories and overarching theme of the result

Meaning units	Codes	Subcategories	Categories	Overachieving theme
It is good if there are experienced RNs on the ward who can help you work out what to do. (FGI 1)	Experienced RNs help NGRNs to find solutions to problems	Support provides security Alone with responsibility for the patient Expectations are not adapted to the competence	Responsibility is not in proportion to the competence	Not being sufficiently prepared and supported to meet responsibilities and demand
You need to keep track of all the prescriptions and know that they are right. (FGI 3)	Need to be sure that medication prescriptions are correct	Difficulty in judging an prescription due to time constraints Afraid to make errors and made errors	Lack of medical competence and experience complicates patient safety	
It's great when you have time to be with patients and see them and you can just go in and keep an eye on them and not have as many patients as we have now. (FGI 2)	Having time to see and be with patients is necessary to be able to provide a good level of nursing care	Need to meet the patient to be able to assess, plan, prioritise lead and distribute nursing care Manage time pressure and getting time for reflections	Strives for control to manage and organise nursing care	

### 3.4 Ethical considerations

The study was given ethical approval by the Ethical Review Board.

## 4 RESULTS

One overarching theme, “Not being sufficiently prepared and supported to meet responsibilities and demands,” and three categories emerged: “Responsibility is not in proportion to competence,” “Lack of medical competence and experience complicates patient safety” and “Strives for control to manage and organise nursing care” (Table 1).

### 4.1 Responsibility is not in proportion to NGRNs' competence

Participants described not having the competence required to be able to individually take responsibility for the nursing caring for complex patient situations. Their competence was described as insufficient, meaning that they required the support of an experienced nurse to help them while managing complex patient situations.

you need colleagues it's really like… how do I manage this situation what should I do… can you show me… (P2, FGI 3)

Experienced RNs are used as a sounding board and help guide NGRNs towards finding solutions to problems. Through the support of experienced colleagues, NGRNs' competence can be monitored before they carry out nursing care in complex patient situations, which gives them a sense of security and safety as well as reducing demands placed on them.

One need to check with experienced nurses … am I think right….you need to get confirmation that you are think right and you need to get the right answers if you have no idea. (P3, FGI 1)

Participants described loneliness, insecurity and lack of support when working shifts with only NGRNs rostered on or where they were the only nurse rostered on, due to the lack of support on offer from experienced RNs when they were working with complex patient situations. These situations often involved NGRNs being made responsible for several complex patient situations simultaneously. When they were working alone and had sole responsibility for patients that they described as difficult, they could experience the situation as chaotic. In these situations, NGRNs seeked out support from either assistant nurses or doctors.

…it can be really hard when there is no one to ask, it is really important that there is someone there who knows more than us and that we are not alone… (P2, FGI 4)

Participating NGRNs described placing high demands on themselves when it came to providing nursing care in several complex patient situations. Expectations were hard to live up to as they are not matched to the NGRNs' competence and could result in them feeling a sense of failure. When expectations from experienced RNs and doctors do not match NGRNs competence, some participants had been criticised for their lack of ability. This criticism was experienced as difficult to take, while deserved, by NGRNs who placed high expectations on themselves but realised they lacked the necessary competence in nursing care for patients in complex situations.

…I hadn't hung the second IV line … they got annoyed with me and rightly so … I was mostly annoyed with myself … I put such high demands on myself … it can break a person if you miss too many things … you just want to cry – how in hell could I have missed that … but I'd been working so hard … (P1, FGI 4)

NGRNs described how they were expected to take responsibility for the introduction of the next group of NGRNs or supervising nursing students with only 6 months of work experience. They could experience the increased responsibility as both stimulating and challenging.

It's an unwritten thing that when you are supervising someone else you take on a whole lot more responsibility … it's good but a lot of responsibility … it's a mixture of fear and fun – being someone who knows more that someone else is scary … you want to be a support for others … but not too early on … no … mmm, I agree. (P3,P4, FGI 2)

They attempt to manage the responsibility, expectations and demands placed on them by pushing themselves and putting their patients' needs first. This could mean that nurses do not find time to eat during a shift or that they would start a shift early so they have time to do everything they need to before rounds start. This created ethical stress that resulted in thoughts about how long they would have the energy to carry on with their jobs.

I don't know how to plan my time because everything seems to happen at once … one night I had so much to do … I fainted because I didn't have time to eat … I just passed out and landed on the floor … it feels stupid that it needs to go so far but the patients always go first …// sometimes I am so tired of caring for others – it's so terrible to say that because that is what we do but I don't have the energy to dress everyone because I hardly have the energy to dress myself … I am thinking about quitting… (P3,P4, FGI 4)

### 4.2 Lack of medical competence and experience complicates patient safety

Participants experienced a lack of medical competence and experience administering, and managing medications safely is a large part of nursing work in complex patient situations, and they do not feel prepared for this. Patients are often prescribed different medications, and this complicates the process of reading up on, reflecting and judging if medications are being administered correctly. The NGRNs try to manage the situations that involve the administration of medication by not stressing, double checking themselves and having a critical approach to prescriptions.

… you are afraid to make mistakes … it needs to go fast … to prepare and connect drip…there is no time to read…it is so stressed you haven't got the time do it right… (P4, FGI 3)

I try to be critical and read … and double check myself…but it is not easy to know if it is right, if I do not know if it is unreasonable then I do not check … then it is very difficult and very dangerous for patients. (P3, FGI 4)

Participating NGRNs described not having time to read carefully or reflect on medication, they sometimes experienced prescriptions as being unclear, and they had difficulty understanding if prescriptions seemed reasonable, which created uncertainty and fear. This meant that they could administer medication without fully understanding the consequences and then think about it afterwards, which increased uncertainty and fear of doing the wrong thing.

… we can have 10–11 patients to one nurse and two assistant nurses on the day shift and one nurse and one assistant nurse on the night shift … you don't have time to see all the patients … all of them have enormous lists of medication (others in groups give signs of agreement). (P3, FGI 2)

NGRNs' lack of confidence and experience as well as shortage of time made the nurses frightened of making mistakes. Their lack of ability could also expose their colleagues to risk, for example by giving patients the wrong medication, which could mean jeopardising patient safety.

… I have given the wrong antibiotics to the wrong patient … another new nurse and I … we gave Tazocin to the same patient ten minutes apart. A doctor prescribed the wrong Midazolam and I administered it by IV instead of injection and the patient just kind of passed out. I didn't know what to do… (P5,FGI 1)

### 4.3 Strives for control to manage and organise nursing care

Participants reported striving for control to manage and organise nursing care in complex patient situations, which dictates that the NGRNs meet patients and participate in their nursing care in order to build a picture of a patient's health status and needs. When the NGRNs were responsible for fewer patients in complex situations and they had control, they felt satisfied and stimulated. When they were responsible for several patients with complex situations, they found it hard to prioritise and described feeling insufficient and that there was never enough time.

It's difficult … if you have several of these complex patients … if it's under control then it's exciting … the complex patients are really difficult and have high demands … to see all their needs, they all have so many… (P 1, FGI 2)

NGRNs needed a detailed report and clear documentation, which improved their possibility to plan and organise care and prioritise patients' needs for care. Meeting patients gives the NGRNs opportunity to judge each patient's needs for nursing care and then have more control over how nursing care is administered.

… you see patients and not just their journals, you have to be there and experience it yourself … even if someone gives you a report it isn't the same thing … how much pain, where and for how long – you have no way of knowing this if you aren't there with the patient. (P 3, FGI 3)

Participants described how difficult it was to find the time required for nursing care for several patients in complex situations and the stress caused results in difficulties in prioritising these patients. Control over nursing care for several patients in complex situations was seen as hard, and participants did not experience that they had the control they needed to do this well. In these complex patients' situations, the NGRNs had lost control and did not know how to structure the nursing care. They described how head of wards could come in and take a leadership role in delegating of the nursing care.

The boss helped prioritize… well … it was so much to have control over…it was infusion pumps, wound dressing… it was so much, different medications and prioritizing after medical round… (P4, FGI 1)

A lack of time meant that basic care was not prioritised and medical needs were taken care of first. This resulted in the NGRNs performing tasks that doctors should perform, and assistant nurses performed tasks that RNs should perform.

You don't have only one patient with comorbidities … the patients have multiple diseases … the ones with the greatest needs are prioritised … a medical patient on the surgery ward and no doctor has met with the patient … it causes more work, you have to keep an eye on things that are not the responsibility of nurses, you take care of those things instead and then the assistant nurses take care of what we nurses should be taking care of… (P2, FGI 3)

They tried to manage their shortage of time by consciously not engaging with the patients or asking about things that were not acute such as lifestyle and existential questions.

… cancer patients need to be asked the existential questions … they often feel terrible and need to talk … if they wake up and find that they lost a leg in an operation and they hadn't been fully informed of the risks beforehand … you need time to properly talk to patients and we just don't have that time… (P1, FGI 4)

Participants described that they did not have control of patients who did not need complex nursing care, those patients simply had to wait and risk getting worse or developing complications. They managed their lack of time by passing on as many duties as possible to the assistant nurses. An experienced assistant nurse was important for the NGRNs to be able to manage their workload in cases where they can trust the assistant nurse's competence.

you are lucky to have an assistant nurse… I just do what I cannot hand over to the assistant nurse and it is enough… you go through in the morning what to do, KAD change, wound, turn the patient, vital parameters… what do I have to do, what is mine, drugs, drip…everything else…but the assistant nurse does not write everything down… they do not have the skills…it happens that patients getting very sick again …and then you are back at square one… (P4, FGI 4)

Unexpected situations often occur with no warning when nursing for patients in complex situations, and the NGRNs find it hard to predict what will happen. They do not have the ability to take control of the situation, which can quickly become unmanageable. This frightens them as they are not prepared for these situations when they occur.

All our patients are sick and things can happen quickly … if something happens you aren't used to or don't know how to handle … the worse thing about being new is when you are faced with situations that you have need been faced with before… (P2, FGI 1)

Reflecting with colleagues at the end of a shift gave the NGRNs the possibility to learn how to do nursing care in complex patients' situations in different ways. It also gave them the possibility to verify how they were thinking, how they handled various situations and to learn from their own and others' experiences.

Having someone to talk to and reflect… is really important… to talk to someone who understands and you learn how to think and do… even if someone says “good work”, you do well anyway… (quiet)… we reflect and then I usually say things like how the day has been and how to do differently… yes… we should do it every day but sometimes just a minute and sometimes you sit down for a while… but it's only on the day shifts… (P 1,2,4, FGI 2)

## 5 DISCUSSION

The aim of the study was to explore NGRNs' experiences and management of complex patient situations. Their experiences and management of complex patient situations can be understood as they are not sufficiently prepared and supported to meet responsibilities and demands as NGRNs.

The participating NGRNs experienced being given responsibilities beyond the scope of their professional competence in complex patient situations without support from experienced RNs. This was also the case when they had to take sole responsibility for the nursing care of several complex patient situations simultaneously. Walker, Costa, Foster, and de Bruin (2017) found that NGRNs' responsibilities in nursing care exceeded their competence. In addition, patients with comorbidities and sudden deterioration were described as challenging and beyond the level of NGRNs' competence (Gellerstedt et al., 2019). A further complexity is when NGRNs solely provide nursing care to patients, the risk of compromising patient safety increases (Murray‐Parahi, DiGiacomo, Jackson, & Davidson, 2016). The limited experience of complex patients' situations among NGRNs (Benner, 2001) can result in them being given a level of responsibility that is too high for them to manage independently. NGRNs can experience complex patients' situations as being more complex due to being task oriented and having difficulties evaluating and prioritising what is important based on a holistic view of each patient's health (Benner, 2001). Hence, boundaries have been pushed over the last decade due to increased patient complexity—patients that previously were treated in intensive care units nowadays often receive care in general hospital settings (Massey, Aitken, & Chaboyer, 2009). It is not surprising that the NGRNs participating in the present study experienced that they needed more support from experienced RNs in handling complex patient situations. Supportive colleagues and constructive feedback are vital for NGRNs in learning to cope with workplace responsibilities (Irwin, Bliss, & Poole, 2018; Lima, Jordan, Kinney, Hamilton, & Newall, 2016). It is, however, surprising and unsuitable for NGRNs to be expected to supervise the induction of other NGRNs after just 6 months of working experience and to be responsible for supervising nursing students' clinical education after even less time. ICN (2013) highlights that healthcare managers should not allow inexperienced NGRNs to practice beyond their level of competence due to concerns regarding patient safety.

In the present study, NGRNs reported a lack of medical competence in judging prescriptions, a fear of making mistakes and actually making mistakes. Medication errors are related to complex patient situations (Saintsing, Gibson, & Pennington, 2011), and NGRNs need further training to better understand the interactions between medications, side effects and the effect of medications on patients' health (Willman, Bjuresäter, & Nilsson, 2020a). It is worrying that participating NGRNs in the current study only referred to the administration of medication but not to side effects, interactions or effects on patient health, which are all competences needed to manage complex patient situations. This highlights the importance of comprehensive nursing education programmes and introduction programmes that successfully prepare NGRNs for clinical practice, as theory and practice can be linked to patients' nursing needs as a starting point.

The NGRNs in the present study did not meet patients as much as they needed due to time shortages and difficulties in managing and organising nursing care for several patients in complex situations. The NGRNs handled this by prioritising other tasks over basic nursing care and avoiding engaging with or talking to patients about their needs. Not being able to meet with patients makes it very hard to accomplish person‐centred care. Due to organisational changes, RNs have more administrative tasks and have moved away from patients, spending less time involved in direct patient care (Scott, Matthews, & Kirwan, 2014). In fact, person‐centred care is important to patient safety, to assure that patients have the information and knowledge they need to act as an advocate for themselves (Bishop & Macdonald, 2017). The results show that the NGRNs' experiences challenge in the leadership and organisation of nursing care in complex patient situations. This is an important finding, and other recent statements (WHO, 2020) and studies presenting results on NGRNs' professional competence (Gardulf et al., 2019; Halabi, Lepp, & Nilsson, 2020; Nilsson et al., 2019) indicate a need to transform nursing education so it has a greater focus on competencies relating to leadership and the organisation of nursing care. These competencies are often taught during the latter part of nursing education programmes (Theander et al., 2016), which will not give nursing students the possibility to internalise theory and practice. Nursing education programmes need to pay more attention to rapidly changing healthcare contexts to prepare nursing students better of their transition into working life.

A lack of experienced RNs and high turnover seem to create a vicious circle as NGRNs participating in this study reported that some shifts were manned by only NGRN, making them solely responsible for several complex patient situations without any support from experienced RNs. When the level of nursing staff is low, quality of care and safety can be compromised, with a risk of substandard nursing care and patient mortality (Aiken et al., 2014; Ball et al., 2018). The main reason for RNs leaving the profession is poor working conditions (SCB, 2017) and unreasonable workloads (Aiken et al., 2012; Rudman & Gustavsson, 2012). To stop this vicious circle of experienced RN shortages affecting the work environment, quality of care and safety, powerful and urgent action is needed to establish sustainable working conditions for RNs. First, hospital leadership and ward management teams need to come up with a specific plan for how to improve working conditions and career paths for all RNs (SCB, 2017). Second, RNs need to hold key leadership positions at all levels of health care to be able to influence working conditions and the organisation of nursing. Third, NGRNs need a good start in the nursing profession by being offered a both generic and individually tailored introduction programme based on NGRNs' need for the further development of their competences in complex patient situations, medication, leading and organising nursing staff and to be supported in a team that includes experienced RNs.

In the present study, NGRNs were forced to push themselves too hard, compromising their health in some cases, to be able to handle complex patient situations. High workloads can lead to high levels of job‐related stress, which in turn can compromise patient safety (Kakemam et al., 2019; Sturm et al., 2019). In the present study, NGRNs performed tasks that should be performed by physicians, and assistant nurses performed tasks that RNs should perform, while ward managers did what experienced RNs should have been doing. A consequence of the insecurity and distress of making errors is increasing occupational stress among RNs (Kakemam et al., 2019). Altogether, nursing care that is not carried out and performing nonprofessional tasks can result in decreased patient safety (Zhang, Li, Guo, & Lee, 2019). In terms of reducing stress levels, developing social support and teamwork has been shown to be an effective strategy (Kakemam et al., 2019; Sturm et al., 2019).

In the process of developing clinical competence that can be applied to complex patient situations, this study showed that reflection gave NGRNs the opportunity to learn how to handle the demands of nursing complex patient situations. NGRNs' clinical competences in acute care hospital settings increased over time, with a significant difference seen between 9–15 months (Willman, Bjuresäter, & Nilsson, 2020b). Experience alone does not automatically lead to increased competence, in order for clinical competence to develop and transit through one competence stage to the next, intellectual and critical thinking is essential (Benner, 2001). Lifelong learning also improves competence (EU, 2013). Taking time to reflect is a dynamic process that involves gaining new perspectives to develop nursing (Davis, Taylor, & Reyes, 2014). In relation to competence development and lifelong learning, the results in this study show the importance of self‐reflection as a way to further develop oneself, and this has also been shown in other studies (Forsman et al., 2019; Nilsson et al., 2019). Yet, the ability to think critically decreased after 9–15 months of clinical practice among NGRNs (Willman et al., 2020b). Possible reasons for a negative development in critical thinking include that NGRNs lack time for reflection, the lack of experienced RNs to support them and give new perspectives and the lack of possibilities for NGRNs to meet patients. This might reduce the opportunities for NGRNs to reflect on how to handle nursing care in complex patient situations.

### 5.1 Strengths and limitations

The strength in FGIs described by Polit and Beck (2021) due to the stimulation that can occur when participants discussing a topic where they have similar experiences, correspond well to the present study, resulted in both individual statements and great interactions. Convenient sampling was chosen and a total of 16 NGRNs participated in the FGIs, which is considered to be a suitable sample size according to Morgan (1997) who suggest to have at least a number of three to four FGIs with 4–6 participants in each FGI (Morgan, 1997), which also will contribute to achieve data saturation. In this study, data saturation was achieved after completing the analysis of the fourth FGI. Limitations to consider were also related to differences between the NGRNs' specialised work units in acute care hospital settings, which may have influenced the results in terms of their differences in experiences related to their reflections. Additional limitations that need to be considered in relation to the transferability of the result are due to the convenience sampling which did not include males, who might have other views and experiences than females. The first author has experience of nursing in complex patient situations, and this preunderstanding may have affected the analysis. Therefor, all authors discussed the preunderstanding thoroughly and have been involved in the analysis process. In addition, the manuscript has been critically discussed in research seminars including the authors and outside researchers on two occasions to accentuate the objectivity of the analysis.

## 6 CONCLUSION

The participating NGRNs were highly influenced by their working environment context where the nursing care in complex patients´ situations took place. NGRNs' experience and management of complex patient situations are related to a lack of support from experienced RNs. They have not been getting adequate support when working with multiple complex patient situations that require a level of competence that surpasses that of NGRNs. There is a need to develop and support nursing leadership at both on individual and organisational level. The RNs and the NGRNs are stretched to the max placing them and the patients at safety risks. To be able to meet the patients regularly during a work shift is the fundament of nursing care and the starting point for reflection, safe management and organised nursing care in complex patient situations. If NGRNs are given sole responsibility for multiple complex patient situations, patient safety may be compromised.

## 7 RELEVANCE TO CLINICAL PRACTICE

Insufficiency competence among NGRNs threatens quality of care and patient safety. NGRNs need support from experienced RNs, especially in regard to complex patient situations that demand more competence in the areas of medical competences, assessing, planning, prioritising, leading and distributing nursing care. A lack of control in managing and organising nursing care will negatively affect person‐centred care. Since the foundation of the profession has not taken responsibility for complex patient situations, this means that introductory programmes, continuous competence development and collegial support are of crucial importance.

## CONFLICT OF INTEREST

The authors declare that they have no conflict of interests.

## Supporting information

File S1
