# Supplementary material for: Insufficiently supported in handling responsibility and demands: Findings from a qualitative study of newly graduated nurses
Source: J Clin Nurs. 2020 Sep 21;30(1-2):83–92. doi: 10.1111/jocn.15483 (PMC7891354; doi:10.1111/jocn.15483)
Supplement: Supplementary file 1 — File S1 [file JOCN-30-83-s001.docx]

**COREQ was used as reporting guidelines in line with EQUATOR (Tong, Sainsbury, & Craig, 2007).**

Consolidated criteria for reporting qualitative studies (COREQ): 32-item checklist.

The page number in the answers are referring to the manuscript.

No Item Guide questions/description

**Domain 1: Research team and reflexivity Personal Characteristics**

**1.Interviewer/facilitator Which author/s conducted the interview or focus group?**

Answer: The first author was the moderator at the FGIs and an assistant moderator (whom are an experienced researcher) was also present taking notes and made a summery in the end of the FGIs.

**2. Credentials What were the researcher’s credentials? E.g. PhD, MD**

Answer: Two of the authors are PhDs and one a PhD-student.

**3. Occupation What was their occupation at the time of the study?**

Answer: Senior lectures at universities and PhD-student.

**4. Gender Was the researcher male or female?**

Answer: Two of the authors are female and one male.

**5. Experience and training What experience or training did the researcher have? Relationship with participants.**

Answer: All of the authors have extensive experience and training in qualitative research methods. The authors had no relationship with the participants.

**6. Relationship established Was a relationship established prior to study commencement?** Answer: Yes. We know each other professionally.

**7. Participant knowledge of the interviewer What did the participants know about the researcher? e.g. personal goals, reasons for doing the research**

Answer: That the study is part of a dissertation. Prior the study they have read an information letter about the aim of the study. The participants has also been given oral information at two separate occasions prior the study and they had the possibility to ask questions.

**8. Interviewer characteristics What characteristics were reported about the interviewer/facilitator? e.g. Bias, assumptions, reasons and interests in the research topic** Answer: In the information letter and orally, it was described how the FGIs would be implemented and whom would be attending. An oral information session were arranged in connection to the NGRNs last mandatory class in nursing education before their graduation. Further, an oral information also took place at the NGRNs first dag at the mandatory clinical development program and prior every FGI. During the oral information sessions, the NGRNs had the opportunity to ask questions to the researchers.

The NGRNs were informed both oral and written that the participation was voluntary with the possibility to withdraw the participation at any time without giving any reason and not elicit negative consequences. The oral and written information contained the aim and a short rationale of the study, methods, the person responsible and contact person for the study. Further, information was given that the research should be used in thesis and publishes as an article.

**Domain 2: study design Theoretical framework**

**9. Methodological orientation and Theory What methodological orientation was stated to underpin the study? e.g. grounded theory, discourse analysis, ethnography, phenomenology, content analysis Participant selection**

Answer: The study has an inductive qualitative design to gain a deeper understanding of the participants´ experience of a phenomenon. Data were analyzed with a text-driven, interpretive qualitative manifest and latent content analysis.

**10. Sampling How were participants selected? e.g. purposive, convenience, consecutive, snowball**

Answer: A convenience sampling was used. The inclusion criteria was to capture NGRNs with six months of working experience as a nurse in an acute care hospital setting.

**11. Method of approach How were participants approached? e.g. face-to-face, telephone, mail, email**

Answer: At the NGRNs first day of the clinical development program an introduction face to face meeting were arranged and the NGRNs who were interested in participating in the study wrote down their contact details, after which they were contacted by telephone by the first author to confirm participation.

**12. Sample size How many participants were in the study?**

Answer: The research group planned to implement a minimum of 4 FGIs with 4-6 participants in each FGI. It was possible to follow out 4 FGIs, thus, in the 4^th^ FGI 2 participant were prevented to participate. Hence, in the present study there were a total of 4 FGIs with 16 participants.

**13. Non-participation How many people refused to participate or dropped out? Reasons? Setting**

Answer: The participants who were interested in participating wrote down their contact details and were then contacted by the first author to confirm the participation and to decide appropriate time and place of the FGI. No one of the participates refused to participate. Two participants dropped out due to it was not possible to arrange schedule with the rest of the participants. There was 20 NGRNs whom wrote down their interest in participating, 18 of them confirmed they wanted to participate in a FGI. Two of them were prevented from participating because the time schedule did not work with the rest of the participants in the FGI. There were a total of 16 participants in 4 FGI that was performed.

**14. Setting of data collection Where was the data collected? e.g. home, clinic, workplace** Answer: At the participants workplace in a pre-boked separate room.

**15. Presence of non-participants Was anyone else present besides the participants and researchers?**

Answer: No

**16. Description of sample What are the important characteristics of the sample? e.g. demographic data, date Data collection**

Answer: Age ranged from 22 to 33 years and the participants had six months of clinical work experience from direct patient care at an acute care hospitals different wards including medical, surgical, emergency, gynecological, psychiatric, and oncology wards.

**17. Interview guide Were questions, prompts, guides provided by the authors? Was it pilot tested?**

Answer: Yes a pilot FGI were arranged that FGI was included in the study. The same semi-structured interview guide was used in every FGI. Prompts was used in all of the FGIs, for example; can you give an example, can you tell more.

**18. Repeat interviews Were repeat interviews carried out? If yes, how many?**

Answer: No.

**19. Audio/visual recording Did the research use audio or visual recording to collect the data?**

Answer: Audio tape

**20. Field notes Were field notes made during and/or after the interview or focus group?** Answer: Yes during the interviews.

**21. Duration What was the duration of the interviews or focus group?**

Answer: From 62 to 75 minutes.

**22. Data saturation Was data saturation discussed?**

Answer: Yes among the research group and the moderators attending at the FGIs. The saturation were discussed during the analysis process, and saturation was assessed to be reached after the analyse of the fourth FGI.

**23. Transcripts returned Were transcripts returned to participants for comment and/or correction?**

Answer: No. However, the assistant moderator made a summary of the interview at the end of the interview and the participant had the opportunity to respond.

**Domain 3: analysis and findings Data analysis**

**24. Number of data coders How many data coders coded the data?**

Answer: Two of the authors made separated codes of two of the interviews. The first author coded the other two.

**25. Description of the coding tree Did authors provide a description of the coding tree?** Answer: Yes. All of the authors has been involved in the analysis process and a coding tree has been provided during this process. The codes and the coding tree are the fundament of the subcategories, the manifest data and the latent data that represent each category.

**26. Derivation of themes Were themes identified in advance or derived from the data?** Answer: Derived from the data. The codes were sorted into subcategories based on differences and similarities, and the three categories were based on the subcategories. From the categories a theme emerge based on latent content.

**27. Software What software, if applicable, was used to manage the data?**

Answer: Not applicable. The authors used their own tables in Word documents to manage data.

**28. Participant checking Did participants provide feedback on the findings? Reporting** Answer: No. However, the analysis process and the results were discussed in two seminars together with several researchers and PhD- students.

**29. Quotations presented Were participant quotations presented to illustrate the themes / findings? Was each quotation identified? e.g. participant number**

Answer: Yes, by the number of participant in the FGI (P) and by the number of the FGI (FGI). For example (P 1, FGI 1).

**30. Data and findings consistent Was there consistency between the data presented and the findings?**

Answer: Yes.

**31. Clarity of major themes Were major themes clearly presented in the findings?**

Answer: Yes, please see Table 1 and the result section.

**32. Clarity of minor themes Is there a description of diverse cases or discussion of minor themes?**

Answer: Yes, as categories.
